# An optimized robotic surgical technique for cervical cancer: investigating whether the use of the pulling robotic arm has better surgical outcomes

**DOI:** 10.3389/fonc.2023.1159081

**Published:** 2023-07-06

**Authors:** Xuzhi Liang, Haijing He, Yingjin Li, Sibang Chen, Jinche Zhao, Bing Yang, Huisi Lin, Hao Zeng, Liuyi Wei, Jiahuang Yang, Jiangtao Fan

**Affiliations:** ^1^ Department of Gynecology, Guangxi Medical University First Affiliated Hospital, Nanning, Guangxi, China; ^2^ Department of Glandular Surgery, The Affiliated Hospital of Youjiang Medical University for Nationalities, Baise, China; ^3^ Department of Gynecology, International Peace Maternal and Child Health Hospital of China Welfare Society, Shanghai, China

**Keywords:** cervical cancer, pulling robotic arm, complication, uterine manipulator, robotic surgery

## Abstract

**Objective:**

The evidence for adopting the 3^rd^ robotic arm (RA) called the pulling RA rather than a uterine manipulator to manipulate the uterus in the robotic radical hysterectomy (RRH) for cervical cancer is still limited. We present a single-center retrospective experience comparing using the pulling RA to replace a uterine manipulator vs. using a uterine manipulator to manipulate the uterus in RRH.

**Methods:**

106 patients diagnosed with IA, IB1-IB2 and IIA1 cervical cancer were retrospectively included for intraoperative and postoperative parameters analysis. 50 patients received RRH by adopting the pulling RA instead of a uterine manipulator to pull the uterus (3-RA RRH group), and another 56 patients were performed RRH with a uterine manipulator (2-RA RRH group). RRH with the pulling RA consisted of a camera arm, 3 RAs including a pulling RA, and 2 conventional assistant arms (3-RA RRH group). In comparison, RRH with a uterine manipulator included 2 RAs and 2 conventional assistant arms (2-RA RRH group). Besides, 3-RA’ RRH group was selected from the 25^th^-50^th^ cases in the 3-RA RRH group based on the learning curve and was compared with the 2-RA RRH group in terms of intraoperative and postoperative parameters.

**Results:**

The patients’ early post-operative complication (≤7 days) (p=0.022) and post-operative anemia (p < 0.001) of the 3-RA RRH were significantly lower than that in the 2-RA RRH group. The results of comparing the 2-RA RRH group with the 3-RA’ RRH group were consistent with the aforementioned results, except for the operative time (220.4 vs. 197.4 minutes, p=0.022) and hospital stay (7.8 vs. 8.7 days, p=0.034). The median follow-up in the 3-RA RRH and 2-RA RRH groups was 29 and 50 months till March 2023. The 3-RA RRH and 2-RA RRH groups’ recurrence rates were 2% (1/50) and 5.4% (3/56), respectively. The mortality in the 3-RA RRH and 2-RA RRH groups was 2% (1/50) and 3.5% (2/56), respectively.

**Conclusion:**

Our study suggested that replacing the uterine manipulator *via* the 3^rd^ RA is viable; the results showed comparable surgical outcomes between the two methods. Thus, 3-RA RRH could be considered a well-executed surgical option in well-selected patients.

## 1 Introduction

According to a worldwide analysis by World Health Organization (WHO), there were an estimated 19.3 million new cases and 10 million deaths of cancer worldwide, of which 604,127 (3.1%) new cases and 341,831 (3.4%) deaths of cervical cancer occurred, and the incidence rate of cervical cancer in 2020 is 13.3/100,000 while the mortality rate was 7.3/100,000 (1). About 450,000 new cases of cervical cancer occurred in developing countries, accounting for 84% of new cases of cervical cancer worldwide (2, 3). The status of radical hysterectomy is unshakable for early and locally advanced cervical cancer therapy (4). Compared with laparoscopic radical hysterectomy (LRH), robot-assisted radical hysterectomy (RRH) has the advantages of less surgical blood loss and short operative time in cervical cancer (5), which has been widely used in the field of gynecological tumors before 2018 (6).

The Laparoscopic Approach to Cervical Cancer (LACC) trial produced a paradigm shift after its publication in October 2018, which showed that the prognosis of patients with minimally invasive surgery (MIS) was significantly worse than that of open abdominal surgery (7). Since the landmark LACC trial, other retrospective studies did come to similar conclusions, such as the retrospective studies of Seoul University in South Korea (8), Canada (9), and the US (10). Therefore, most global guidelines or consensus have proposed that radical hysterectomy for cervical cancer requires careful selection of MIS, including robotic surgery. However, the notable criticism against the LACC trial was that the participating surgeons were required to provide only ten minimally invasive radical hysterectomies and two videos to prove technical competency. A relevant study proved that surgeons were still in the early stages of the learning curve after completing 10 MISs, which was associated with poorer outcomes (11). In addition, another criticism against the LACC trial was that the robotic-assisted radical hysterectomy (RRH) accounted for a low proportion (15.6%), while the MEMORY study, a multi-institutional retrospective analysis, had a large percentage (78%) of patients who underwent robotic-assisted procedures (12). Interestingly, the MEMORY study revealed that MIS compared to abdominal radical hysterectomy (ARH) for cervical cancer did not jeopardize oncologic outcomes, including progression-free survival (PFS) and overall survival (OS). Moreover, another retrospective study came up with the opposite conclusion, arguing that OS was higher in the MIS group compared with the ARH (13). Although the above studies cannot be of equivalent quality to the LACC trial, it is believed that MIS for cervical cancer in selected cases (tumor diameters less than 2 cm) will not affect the oncologic outcomes (14, 15).

Although it is known that MIS for malignant tumors also needs to avoid iatrogenic tumor implantation at the initial treatment, it is more difficult to follow that principle under the laparoscopic situation. The reasons are as follows: (1) the laparoscope increases the opportunity for tumor cell exfoliation; (2) repeated contact between the medical instrument contaminated with tumor cells and the piercing point; and (3) exfoliation of the malignant cells while taking out the excised tissue at the point of punctuation (16). Based on the conclusions of the LACC study, MIS for cervical cancer without a uterine manipulator and performing vaginal ligation below the lesion of cervical cancer and cutting off the vagina below the ligation line are now standard. Nevertheless, the method to control the uterus without a uterine manipulator is concerning. Researchers are trying to improve surgery methods, such as the non-touch isolation technique (without using a uterine manipulator and cutting off vagina below the ligation line) (17–19), which is gradually being tried in clinical practice. As we gained more experience, we introduced an improved technique using an additional robotic arm (RA) to replace the uterine manipulator and investigated its utility in robotic radical hysterectomy (RRH). However, the study investigating the strategy of three-robotic arms RRH (3-RA RRH) without a uterine manipulator is still limited.

Herein, we hypothesize that the improved strategy of the 3^rd^ RA replacing the uterine manipulator and cutting off vagina below the ligation line can improve the surgical outcomes of RRH for early cervical cancer. However, research addressing the surgical outcomes of 3-RA RRH is lacking since 3-RA RRH is an optimized operation approach, and its surgical results have not been explored in randomized controlled trials. Therefore, our study aimed to compare the short-term surgical results, including operative time, blood loss, drainage of day 1, 2, and 3, anal exhaust time, and postoperative complications of 3-RA and 2-RA RRH.

## 2 Materials and methods

### 2.1 Patients

Our study obeyed the presentation of Strengthening the Reporting of Observational Studies in Epidemiology (20). The study was conducted in accordance with the Declaration of Helsinki and was approved by the ethics committee of our hospital. 106 patients were carried out RRH with pelvic lymphadenectomy in our center from January 2018 to May 2021. Patients were informed of the different treatment options’ pros and cons. Also, after the publication of the LACC research, we informed the later patients of the results’ data. The choice of surgical method mainly depended on the patient’s economic factors and the complexity of the condition, and was evaluated by the surgeon before surgery. Before operations, all patients signed the consent to undergo laparoscopic surgery using the da Vinci Si^®^ Surgical System at Guangxi Medical University First Affiliated Hospital.

The clinical stages of patients were sorted based on the International Federation of Gynecology and Obstetrics (FIGO) classification modified in 2018 (21). Inclusion criteria were: (1) age greater than 18 years; (2) patients with newly diagnosed and previously untreated cervical cancer; (3) FIGO stage IA, IB1-IB2 and IIA1; and (4) patients with complete medical records.

Excluding criteria were: (1) large uteri (≥500g) (22); (2) pregnancy; (3) women who were with other tumors or severe co-morbidities and/or had pneumoperitoneum contraindication; (4) the preoperative examination suggested anemia, hypoproteinemia, urinary retention, urinary tract infection, sepsis, pelvic collection, dynamic ileum, pyrexia, lower limb hyposthenia, bowel subocclusion, dysuria, ureteral fistula, ureteral stenosis, hydronephrosis or venous thrombosis.

A complete physical and rectovaginal examination was performed by the surgeon before the operation. In addition, accurate clinical staging of cervical cancer also required chest radiography and transvaginal ultrasound. The vast majority of cases had abdominal and pelvic MRI or CT. Experimental objects were divided into 3-RA RRH and 2-RA RRH groups based on the type of surgical method. The 3-RA RRH and 2-RA RRH procedures were performed by the same surgical gynecologist and operation team.

### 2.2 Surgical techniques

All the procedures were conducted by da Vinci Si^®^ Surgical System (Intuitive Surgical). The patient should be changed into a steep Trendelenburg position and dorsal lithotomy position with adequate sacral support after general anesthesia, and then urinary bladder catheterization was performed. After creating pneumoperitoneum, ports were placed by Hasson approach at the umbilicus (23). Radical hysterectomy with removal of bilateral pelvic lymph nodes was included in the surgical management of cervical cancer. The decision to perform paraaortic lymph node dissection depended on the intraoperative investigation. A radical hysterectomy was conducted and the procedure included seven parts: (1) bilateral pelvic lymphadenectomy; (2) sufficiently expanding the spaces of paravesical and pararectal; (3) ureteral dissection; (4) ligation and dissection of the uterine artery; (5) fully expanding the spaces of vesicouterine and rectovaginal; (6) resection of the parametria, and (7) resection of the upper vagina.

### 2.3 Robot-assist radical hysterectomy

**Figure 1:**
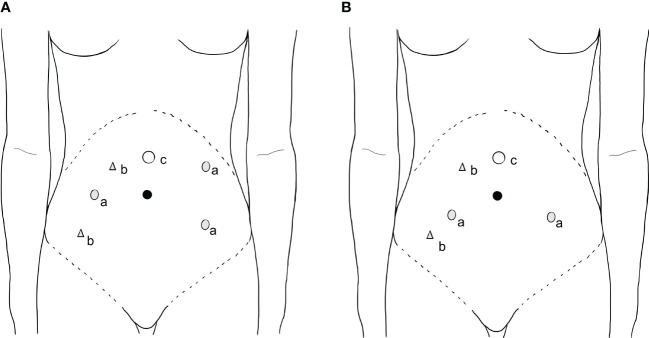
Port placement. (a) **(A)** (3-RA RRH): Three robotic 8-mm trocars were placed: 1^st^ RA was 6 cm right side parallel and level to the umbilicus; 2^nd^ RA was 6 cm left side and below the umbilicus; 3^rd^ RA (also named as pulling arm) was 6 cm left side and 6 cm above of the 2^nd^ RA, just near the left arch of rib. **(B)** (2-RA RRH): two robotic 8 mm trocars were introduced in each lower quadrant of the abdomen, lateral to the epigastric arteries, 2 to 3 cm below the umbilical level (1^st^ and 2^nd^ RA). (b) The assistant 10mm trocar was placed 2 cm inside the right anterior superior spine. Another 5-mm assistant trocar was placed in the middle and 1 cm outside the camera and 1^st^ RA if needed. (c) The 12-mm camera port was placed 4-5cm right above or left-of-middle the umbilicus depending on the size of the uterus. RA: robotic arm, 2-RA RRH: two-robotic arms radical robot-assisted hysterectomy, 3-RA RRH: three-robotic arms radical robot-assisted hysterectomy.

**Figure 2:**
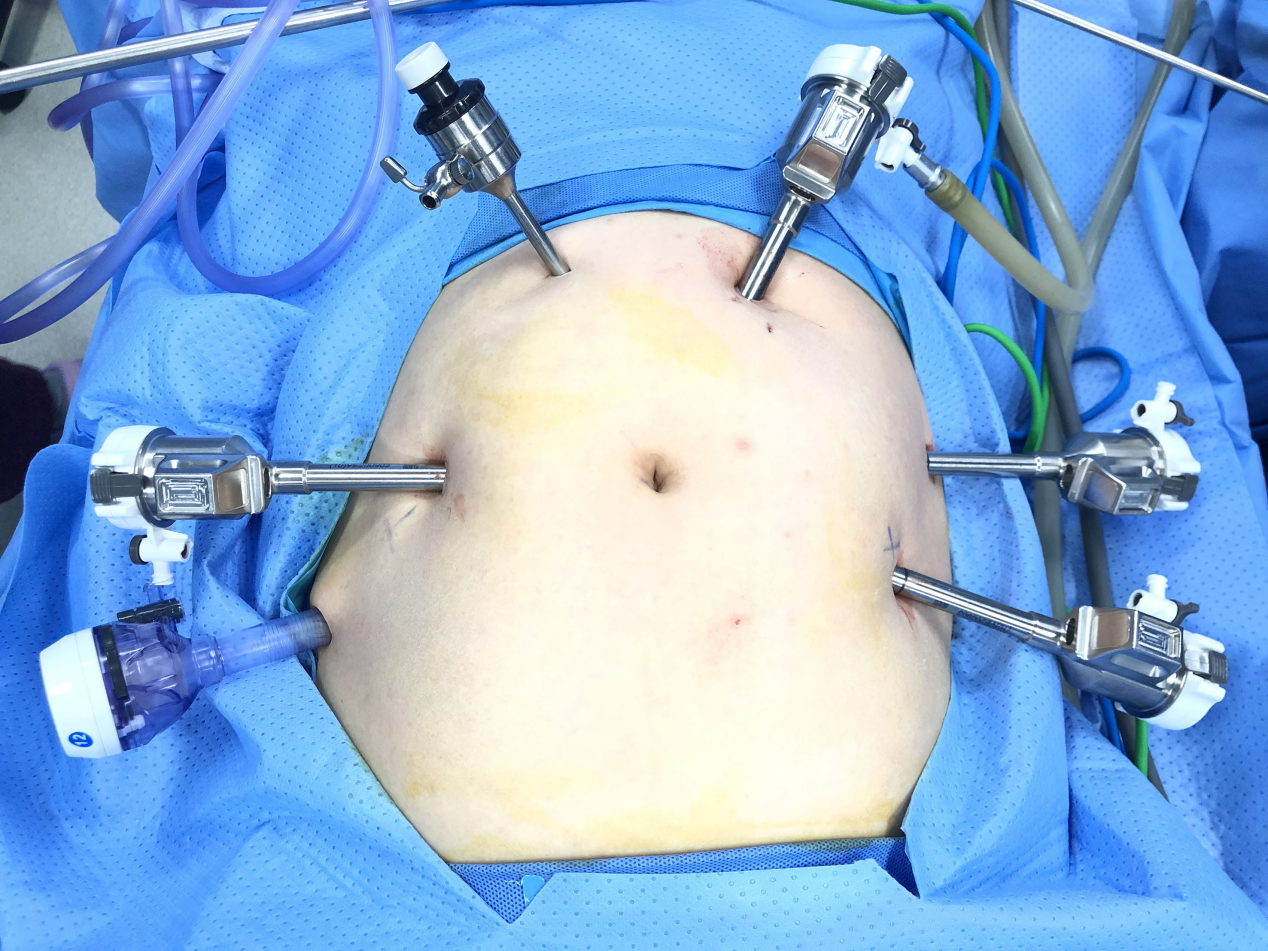
Photograph of the placement of ports in the 3-RA RRH group. 3-RA RRH: three-robotic arms radical robot-assisted hysterectomy.

Port placement and operating procedures for RRH were performed as previously reported (24). An optimized technique of our procedure in the 3-RA RRH group was the utilization of the pulling RA (3^rd^ RA), a substitute for a uterine manipulator to manipulate the uterus, and the performance of vaginal ligation below the lesion of cervical cancer and cutting off the vagina below the ligation line. The 3^rd^ RA was an atraumatic forceps (Intuitive Surgical 420049 Cadiere Forceps, 8mm) used to clamp the uterine horn and maintain the tension of the uterus to optimize the surgical field. In the 3-RA RRH group, three robotic 8-mm trocars were placed: 1^st^ RA was 6 cm right side parallel and level to the umbilicus; 2^nd^ RA was 6 cm left side and below the umbilicus; 3^rd^ RA was 6 cm left side and 6 cm above of the 2^nd^ RA, just near the left arch of rib. Surgery was generally performed with one assistant port (10mm). The need for an additional assistant port depends on the intraoperative situation. If serious adhesion occurs between the uterus and the surrounding tissues and organs during the operation, another 5-mm assistant port may be placed (**Figures 1A**, **2**). The following instruments, including a bipolar grasper as well as a PK grasper on the left and right upside and robotic trocars (1^st^ and 2^nd^ RAs, respectively) were introduced, and the 3^rd^ RA, an atraumatic forceps on the left robotic trocar. In the 2-RA RRH group using a uterine manipulator, two 8-mm trocars were placed in the bilateral lower quadrant, lateral to the epigastric arteries, 2 to 3 cm below the umbilical level (1^st^ and 2^nd^ RAs, respectively). The assistant trocars were the same as in the 3-RA RRH group (**Figure 1B**).

### 2.4 Clinical parameters and outcomes

The total operative time was subdivided as follows and has defied in the previous study (25): (1) preparation time; (2) docking time; (3) console time; and (4) closure time. Intraoperative parameters included estimated blood loss and operative time. Postoperative parameters included length of hospital stay; drainage of day 1, 2 and 3; early postoperative complication (≤7 days): post-operative anemia (hemoglobin concentration below 7.5 mmol/L (12 g/dL) (26), post-operative hypoproteinemia (albumin concentration below 35 g/L) (27), urinary retention, urinary tract infection, sepsis, pelvic collection, dynamic ileum, pyrexia, lower limb hyposthenia, bowel subocclusion; and postoperative complication after >7 days: dysuria, lymphocele, lymphedema, lymphorrhea, ureteral fistula, vesico-vaginal fistula, ureteral stenosis, hydronephrosis, venous thrombosis, poor healing of vaginal cuff, abdominal wound dehiscence. This complication has been adopted by a previous study (28). Disease-free survival (DFS) was defined as the duration from the date of initial diagnosis to the date of recurrence based on imaging findings, tissue biopsy, or the date of the last follow-up. OS was defined as the duration from the ate of initial diagnosis to the date of cancer-related death or the last follow-up.

### 2.5 Statistics

Shapiro-Wilk (W test) was used to test the normality of continuous variables. If the data conformed to the normal distribution, the independent-sample t-test was used, and if it did not, the non-parametric test was used. The independent-sample t-test or Mann-Whitney was utilized to compare continuous variables, while chi-square or Fisher’s test analysis was utilized to compare binary variables. P-value < 0.05 or adjusted p-value < 0.05 was regarded as statistically significant. The learning curve was obtained by evaluating consecutive cases using the cumulative sum (CUSUM) method. The statistical analyses were conducted *via* IBM SPSS version 22.0 (SPSS Inc, Chicago, IL, USA).

### 2.6 Ethical statements

The study was approved by the Research Ethics Committee of Guangxi Medical University First Affiliated Hospital 2021(KY-E-176).

## 3 Results

### 3.1 Patient characteristics

In total, 106 patients were identified between January 2018 and May 2021. The demographic and clinicopathological information was presented in **Table 1**. 3-RA RRH was conducted in 50 patients (47.2%), while 56 patients (52.8%) underwent 2-RA RRH. No conversions to open laparotomy occurred in the subjects. There was no difference in most of the subjects’ baseline information, excepted for the age and number of pelvic nodes removed (**Table 1**).

**Table 1:** Baseline characteristics of the enrolled patients.

Characteristics	3-RA RRH (n=50)	2-RA RRH (n=56)	p-value
Age (yr)	51.3 ± 9.4	47.3 ± 9.4	0.032^a^
BMI (kg/m2)	22.7 ± 2.9	22.9 ± 4.3	0.685^a^
HPV			0.337^b^
Yes	35 (70.0)	32 (57.1)	
No	5 (10.0)	6 (10.7)	
NA	10 (20.0)	18 (32.1)	
Stage			0.518^c^
IA	0	2 (3.6)	
IB1-2	42 (84.0)	47 (83.9)	
IIA1	8 (16.0)	7 (12.5)	
Grading			0.679^b^
1	15 (30.0)	16 (28.6)	
2	26 (52.0)	33 (58.9)	
3	9 (18.0)	7 (12.5)	
Histology			0.117^c^
Squamous	36 (72.0)	46 (82.1)	
Adenocarcinoma	11 (22.0)	6 (10.7)	
Squamous and adenocarcinoma	1 (2.0)	4 (7.1)	
Others	1 (2.0)	0	
Myometrial invasion			0.060^c^
None	8 (16.0)	9 (16.1)	
<1/3	10 (20.0)	6 (10.7)	
1/3-2/3	21 (42.0)	38 (67.9)	
>2/3	11 (22.0)	3 (5.4)	
Lymphovascular space involvement	12 (24)	22 (40.4)	0.092^b^
No. of pelvic nodes removed	17 ± 7	14 ± 7	0.029^a^
Positive pelvic nodes	7 (13.2)	8 (14.0)	0.996^b^
Neoadjuvant chemotherapy	3 (6.0)	3 (5.4)	>0.999^c^
Adjuvant therapy	33 (66.0)	32 (57.1)	0.350^b^

Values are presented as mean ± standard deviation or percentage (%). a: t-test, b: Chi-square test, c: Fisher’s test.

NA, not available; 2-RA RRH, two-robotic arms radical robot-assisted hysterectomy; 3-RA RRH, three-robotic arms radical robot-assisted hysterectomy.

### 3.2 Surgical outcomes data of the enrolled patients

The operation outcomes data were displayed in **Table 2**. The estimated blood loss was 68.7 ± 55.4 and 78.8 ± 60.7 ml in the 3-RA and 2-RA RRH groups, respectively. No significant difference was found in the blood loss between the two groups (p=0.373). The operative time, length of hospitalization, drainage of day 1, 2 and 3 and anal exhaust time did not differ between the two groups.

**Table 2:** Surgical outcomes data of the enrolled patients.

Outcomes	3-RA RRH (n=50)	2-RA RRH (n=56)	p-value
Operative time (min)	218.9 ± 46.2	220.4 ± 42.6	0.863
Blood loss (mL)	68.7 ± 55.4	78.8 ± 60.7	0.373
Hospital stay (days)	8.7 ± 2.0	8.7 ± 2.0	0.993
Day1 drainage (mL)	148.7 ± 112.5	127.7 ± 156.5	0.433
Day2 drainage (mL)	103.4 ± 123.4	125.9 ± 166.8	0.437
Day3 drainage (mL)	70.4 ± 128.6	54.7 ± 93.2	0.469
Anal exhaust time (days)	1.9 ± 0.5	2.0 ± 0.6	0.471

Values are presented as mean ± standard deviation. The differences between the two groups were tested via t-test.

2-RA RRH, two-robotic arms radical robot-assisted hysterectomy; 3-RA RRH, three-robotic arms radical robot-assisted hysterectomy.

### 3.3 Complications

Postoperative complications (≤7 days) after intervention were shown in **Table 3**. The overall incidence of postoperative complications within 7 days was 44% (22/50) in the 3-RA RRH group and 66.1% (37/56) in the 2-RA RRH group (p=0.022). In the 3-RA RRH group, postoperative anemia within 7 days occurred in 16.0% (8/50) and patients 48.2% (27/56) in the 2-RA RRH group, which showed a significant difference (p<0.001). The rate of post-operative hypoproteinemia was higher in the 2-RA RRH group, although without a significant difference (p=0.573).

**Table 3:** Patients with the complication of early postoperative (≤7 days) of the 3-RA RRH and 2-RA RRH groups.

Complications	3 -RA RRH (n=50)	2-RA RRH (n=56)	p-value
Early post-operative complication (within 7 days)	22 (44.0)	37 (66.1)	0.022^a^
Post-operative anemia	8 (16.0)	27 (48.2)	<0.001^a^
Post-operative hypoproteinemia	17 (34.0)	22 (39.3)	0.573^a^
Urinary retention	0	1 (1.8)	>0.999^b^
Urinary tract infection	0	2 (3.6)	0.497^b^
Sepsis	0	0	>0.999^b^
Pelvic collection	0	1 (1.8)	>0.999^b^
Dynamic ileum	0	0	>0.999^b^
Pyrexia	3 (6.0)	5 (8.9)	0.720^b^
Lower limb hyposthenia	0	0	>0.999^b^
Bowel subocclusion	1 (2.0)	5 (8.9)	0.210^b^

Values are presented as percentage (%). a: Chi-square test, b: Fisher’s test.

2-RA RRH, two-robotic arms radical robot-assisted hysterectomy; 3-RA RRH, three-robotic arms radical robot-assisted hysterectomy.

We detected no significant difference in the postoperative complication after >7 days between the two groups (**Table 4**). A larger proportion (9/56) of patients in the 2-RA RRH group were prone to occur postoperative complications after >7 days compared with the 3-RA RRH group (4/50) without significant difference. Hydronephrosis occurred in 2 patients in the 3-RA RRH group and 3 patients in the 2-RA RRH group (p>0.999). 3 patients in the 3-RA RRH group and 4 patients in the 2-RA RRH group were diagnosed with dysuria after > 7 days (p>0.999). Stent placement for 3 months was adequate to recover the ureteral integrity for all cases. One patient had a vesico-vaginal fistula and ureteral fistula in the 3-RA RRH group, and underwent conservative treatment with an indwelling Foley catheter in the bladder for 2 weeks.

**Table 4:** Patients with postoperative complication after >7 days of the 3-RA RRH and 2-RA RRH groups.

Complications	3-RA RRH (n=50)	2-RA RRH (n=56)	p-value
Post-operative complication after >7 days	4 (8.0)	9 (16.1)	0.206^a^
Dysuria	3 (6.0)	4 (7.1)	>0.999^b^
Lymphocele	0	0	>0.999^b^
Lymphedema	0	0	>0.999^b^
Lymphorrhea	0	1 (1.8)	>0.999^b^
Ureteral fistula	1 (2.0)	0	0.472^b^
Vesico-vaginal fistula	1 (2.0)	0	0.472^b^
Ureteral stenosis	1 (2.0)	0	0.472^b^
Hydronephrosis	2 (4.0)	3 (5.4)	>0.999^b^
Venous thrombosis	0	0	>0.999^b^
Poor healing of vaginal cuff	0	1 (1.8)	>0.999^b^
Abdominal wound dehiscence	0	0	>0.999^b^

Values are presented as percentage (%). a: Chi-square test, b: Fisher’s test.

2-RA RRH, two-robotic arms radical robot-assisted hysterectomy; 3-RA RRH, three-robotic arms radical robot-assisted hysterectomy.

### 3.4 Comparison between 3-RA’ RRH group and 2-RA RRH group

**Figure 3:**
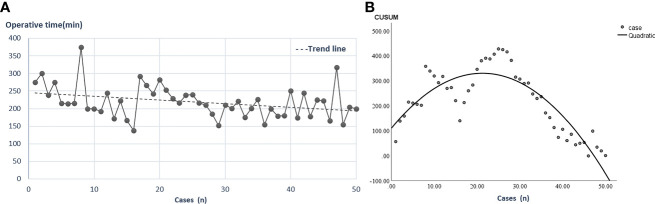
Operative time trending and learning curve in the 3-RA RRH group. **(A)**: The relationship between the number of cases and operative time in the 3-RA RRH group. **(B)**: CUSUM learning curve, the 24^th^ case spanning the learning curve vertices. 3-RA RRH: three- robotic arms radical robot-assisted hysterectomy, CUSUM: cumulative sum.

Since the 2-RA RRH was started in our center in 2016, while the 3-RA RRH was performed later in January 2020, the surgeon was more proficient with the 2-RA RRH technique compared with 3-RA RRH. Thus, it was not sufficient enough to directly conclude that 3-RA RRH was superior to 2-RA RRH for the items in which significant differences were found. The operative time trending in the 3-RA RRH group showed that the required operative time decreased as the number of cases increased (**Figure 3A**). The CUSUM learning curve was calculated by SPSS 22.0 software, and the quadratic curve (R^2 =^ 0.945, p<0.002) was the best-fitting model. The fitting equation is CUSUM (min)=-0.487x^2 +^ 20.700x+109.876 (x is the number of cases). The curve across the apex when the cases accumulate to the 24^th^ case. The learning curve can be divided into two stages according to the apex of the 24^th^ case, including stages A and B. Stage A is the learning improvement stage, and stage B is the proficiency stage (**Figure 3B**). The 24^th^ case in the 3-RA group was taken as the threshold value, and the latter 26 cases in the 3-RA RRH group were selected as the 3-RA’ RRH group to compare with the 2-RA RRH group.

The clinicopathological features characteristics of 2-RA and 3-RA’ RRH were shown in detail in **Table 5**. No difference was found in most baseline characteristics, excepted for age, the number of pelvic nodes removed, and lymphovascular space involvement (LVSI). The results data revealed that significant differences favoring 3-RA’ RRH group were demonstrated for operative time (197.4 vs. 220.4 mins, P=0.022) and hospital stay (7.8 vs. 8.7 days, p=0.034) compared to 2-RA RRH group (**Table 6**). Postoperative complications (≤7 days) of 2-RA and 3-RA’ RRH groups were shown in **Table 7**. The overall incidence of postoperative complications within 7 days of the 2-RA RRH group was significantly higher than that in the 3-RA’ RRH group (66.1% VS. 38.5%, p=0.019), in which the incidence of postoperative anemia was lower in the 3-RA’ RRH group with significance (p=0.031). **Table 8** displayed no significant difference in the postoperative complication after >7 days between the two groups.

**Table 5:** Baseline characteristics of the 3-RA’ RRH and 2-RA RRH groups.

Characteristics	3-RA’ RRH (n=26)	2-RA RRH (n=56)	p-value
Age (yr)	53.1 ± 9.5	47.3 ± 9.4	0.012^a^
BMI (kg/m2)	22.4 ± 2.1	22.9 ± 4.3	0. 529^a^
HPV			0.101^c^
Yes	18 (69.2)	32 (57.1)	
No	5 (19.2)	6 (10.7)	
NA	3 (11.5)	18 (32.1)	
Stage			>0.999^c^
IA	0	2 (3.6)	
IB1-2	21 (80.8)	47 (83.9)	
IIA1	5 (19.2)	7 (12.5)	
Grading			0.686^b^
1	6 (23.1)	16 (28.6)	
2	15 (57.7)	33 (58.9)	
3	5 (19.2)	7 (12.5)	
Histology			0.311^c^
Squamous	22 (84.6)	46 (82.1)	
Adenocarcinoma	3 (11.5)	6 (10.7)	
Squamous and adenocarcinoma	0	4 (7.1)	
Others	1 (3.8)	0	
Myometrial invasion			0.995^c^
None	4 (15.4)	9 (16.1)	
<1/3	7 (26.9)	6 (10.7)	
1/3-2/3	9 (34.6)	38 (67.9)	
>2/3	6 (23.1)	3 (5.4)	
Lymphovascular space involvement	12 (46.2)	22 (40.4)	0.03^b^
No. of pelvic nodes removed	18 ± 8	14 ± 7	0.025^a^
Positive pelvic nodes	5 (19.2)	8 (14.0)	0.746^c^
Neoadjuvant chemotherapy	1 (3.8)	3 (5.4)	>0.999^c^
Adjuvant therapy	16 (61.5)	32 (57.1)	0.811^c^

Values are presented as mean ± standard deviation or percentage (%). a: t-test, b: Chi-square test, c: Fisher’s test.

NA, not available; 2-RA RRHm two-robotic arms radical robot-assisted hysterectomy; 3-RA’ RRH; three-robotic arms’ radical robot-assisted hysterectomy (3-RA’ RRH group was selected from the 25th-50th cases in the 3-RA RRH group based on the learning curve).

**Table 6:** Surgical outcomes data of the 3-RA’ RRH and 2-RA RRH groups.

Outcomes	3-RA’ RRH (n=26)	2-RA RRH (n=56)	p-value
Operative time(min)	197.4 ± 38.6	220.4 ± 42.6	0.022
Blood loss (mL)	65.4 ± 64.9	78.8 ± 60.7	0.364
Hospital stay (days)	7.8 ± 1.4	8.7 ± 2.0	0.034
Day1 drainage(mL)	148.7 ± 112.5	127.7 ± 156.5	0.714
Day2 drainage(mL)	103.4 ± 123.4	125.9 ± 166.8	0.384
Day3 drainage(mL)	70.4 ± 128.6	54.7 ± 93.2	0.613
Anal exhaust time (days)	1.8 ± 0.6	2.0 ± 0.6	0.068

Values are presented as mean ± standard deviation. The differences between the two groups were tested via Mann–Whitney Ut test. 2-RA RRH, two-robotic arms radical robot-assisted hysterectomy; 3-RA’ RRH, three-robotic arms radical robot-assisted hysterectomy (3-RA’ RRH group was selected from the 25th-50th cases in the 3-RA RRH group based on the learning curve).

**Table 7:** Patients with the complication of early postoperative (≤ 7 days) of the 3-RA’ RRH and 2-RA RRH groups.

Complications	3 -RA’ RRH (n=26)	2-RA RRH (n=56)	p-value
Early post-operative complication (within 7 days)	10 (38.5)	37 (66.1)	0.019^a^
Post-operative anemia	6 (23.1)	27 (48.2)	0.031^a^
Post-operative hypoproteinemia	5 (19.2)	22 (39.3)	0.072^a^
Urinary retention	0	1 (1.8)	>0.999^b^
Urinary tract infection	0	2 (3.6)	>0.999^b^
Sepsis	0	0	>0.999^b^
Pelvic collection	0	1 (1.8)	>0.999^b^
Dynamic ileum	0	0	>0.999^b^
Pyrexia	3 (11.5)	5 (8.9)	0.704^b^
Lower limb hyposthenia	0	0	>0.999^b^
Bowel subocclusion	1 (3.8)	5 (8.9)	0.659^b^

Values are presented as percentage (%). a: Chi-square test, b: Fisher’s test.

2-RA RRH, two-robotic arms radical robot-assisted hysterectomy; 3-RA’ RRH, three-robotic arms radical robot-assisted hysterectomy (3-RA’ RRH group was selected from the 25th-50th cases in the 3-RA RRH group based on the learning curve).

**Table 8:** Patients with postoperative complication after >7 days of the 3-RA’ RRH and 2-RA RRH groups.

Complications	3-RA’ RRH (n=26)	2-RA RRH (n=56)	p-value
Post-operative complication after >7 days	3 (11.5)	9 (16.1)	0.744^b^
Dysuria	2 (7.7)	4 (7.1)	>0.999^b^
Lymphocele	0	0	>0.999^b^
Lymphedema	0	0	>0.999^b^
Lymphorrhea	0	1 (1.8)	>0.999^b^
Ureteral fistula	1 (3.8)	0	0.317^b^
Vesico-vaginal fistula	1 (3.8)	0	0.317^b^
Ureteral stenosis	1 (3.8)	0	0.317^b^
Hydronephrosis	2 (7.7)	3 (5.4)	0.650^b^
Venous thrombosis	0	0	>0.999^b^
Poor healing of vaginal cuff	0	1 (1.8)	>0.999^b^
Abdominal wound dehiscence	0	0	>0.999^b^

Values are presented as percentage (%). a: Chi-square test,b: Fisher’s test.

2-RA RRH, two-robotic arms radical robot-assisted hysterectomy; 3-RA’ RRH, three-robotic arms radical robot-assisted hysterectomy (3-RA’ RRH group was selected from the 25th-50th cases in the 3-RA RRH group based on the learning curve).

### 3.5 Oncologic survival outcomes

The majority of cases (35/50) in the 3-RA RRH group did not reach three years after initial treatment. Thus, the 3-year or 5-year survival analysis was not viable now. The more accessible mortality and recurrence rates were obtained as follows. The median follow-up in the 3-RA RRH and 2-RA RRH groups was 29 and 50 months till March 2023, respectively. In the 3-RA RRH group, one case died of thrombocytopenia and pulmonary hemorrhage but was not attributable to cervical cancer; one case (stage IIA1) relapsed with disease-free survival (DFS) of 22 months but is alive with the recorded disease. The recurrence rate in the 3-RA RRH group was 2% (1/50). In the 2-RA RRH group, one case died of systemic metastasis after recurrence (stage IB1), whose DFS was 31 months, and one died of systemic metastasis after recurrence (stage IB1), whose DFS was unclear. The OS was 33 and 36 months, respectively. Moreover, one case (stage IB1) relapsed with DFS of 40 months but is alive with the recorded disease. The mortality in the 2-RA RRH group was 3.5% (2/56), and the recurrence rate was 5.4% (3/56).

## 4 Discussion

Most guidelines for cervical cancer stated that open surgery was the standard surgical method for radical hysterectomy in early cervical cancer. However, controversies still exist. The SUCCOR (Surgery in Cervical Cancer, Observational, Retrospective) study showed that for early cervical cancer, the recurrence rate of patients who performed MIS with a uterine manipulator was 2.76-times that of patients who underwent ARH, and there was no difference in the recurrence rate between MIS with protective vaginal closure and ARH. What’s more, the recurrence rate in the MIS with protective vaginal closure was not different from that in the ARH (29). The results suggested that inferior survival of early-stage cervical cancer in the MIS of the LACC trial may not be due to the MIS technique itself but rather to technical irrationalities, such as the use of a uterine manipulator and unprotected vaginal amputation. Thus, researchers are encouraged to actively explore and improve the details of the surgery, such as avoiding the use of a uterine manipulator and improving the method of vaginal resection, etc. (19, 30). Based on previous research and clinical practice, we used the 3^rd^ RA (pulling RA) rather than the uterine manipulator to manipulate the uterus, as well as cutting off the vagina below the cervical lesion before colpotomy.

The present study obtained surgical outcomes of 3-RA RRH and 2-RA RRH in the therapy of 106 cervical cancer patients. The main finding of our study was the probability of complications within 7 days after surgery of the 3-RA RRH group was significantly lower than that of the 2-RA RRH group. Regarding postoperative complications, there was no significant difference in the 3-RA RRH and the 2-RA RRH group except for post-operative anemia. The probability of postoperative anemia in the 2-RA RRH group (27/56) was significantly higher than that of the 3-RA RRH group (8/50). More estimated blood loss in the 2-RA RRH group (68.7 mL and 78.8 mL in the 3-RA RRH and 2-RA RRH groups, respectively) may lead to this result, although there was no statistical significance. Also, when adequate tissue traction is performed with a gripper in the 3^rd^ RA, precise tissue dissection can be steadily performed, reducing bleeding to nonessential conditions. In addition, no difference was found in operative time between 3-RA RRH and 2-RA RRH groups (218.9 vs. 220.4min p=0.863). However, after omitting the former 24 cases of the 3-RA RRH group based on the learning curve and comparing the 3-RA’ RRH group with the 2-RA RRH group, the operative time of the 3-RA’ RRH group was significantly shorter than the 2-RA RRH group (197.4 vs. 220.4 min, p=0.022). In our center, using a uterine manipulator in 2-RA RRH has been the standard surgical method since the primary RRH surgery was carried out. Nevertheless, the technique of utilizing 3^rd^ RA to pull the uterus without the uterine manipulator was only adopted for two years, which may explain why we observed no difference in the operative time between 3-RA RRH and 2-RA RRH groups. Therefore, we assume that 3-RA RRH can reach a relatively skilled and stable level after 24^th^ cases, and achieve a shorter operative time and more satisfactory surgical outcome.

Using a uterine manipulator inevitably increases intrauterine pressure. It pushes tumor cells beyond the myometrial barrier or even into the abdominal cavity through the fallopian tube, leading to the spread, planting, and metastasis of tumor cells. Also, the inherent and limited adjustment angle of a uterine manipulator brings a challenge in adjusting the position of the uterus to expose the surgical field of some deep positions, which causes compression and damage to the parauterine tissues and adjacent organs. The compression of the endometrium caused by a uterine manipulator not only leads to the injury of the endometrium but also causes ischemic endometrium necrosis, which results in the activation of the stress response system, causing harm to the tissue to a certain extent. A cohort observational study that included 1272 stage lB1 cervical cancer patients by Chiva et al. (29) proposed that using a uterine manipulator jeopardized OS in patients who underwent MIS. Thus, some surgeons avoid using a uterine manipulator because of concerns about changing the pathology or inducing LVSI. However, studies by Rakowski et al. (31) and Liu et al. (32) showed that the use of a uterine manipulator did not seem to change the risk of recurrence. What’s more, some researchers have also indicated that the use of a uterine manipulator in patients with stages IA1-IIA cervical cancer undergoing MIS was not an independent factor related to the recurrence rate (33), so it is unlikely to affect the treatment effect.

Despite whether manipulation of the uterus without a uterine manipulator improves outcomes is inconclusive, there are still other novel explorations to avoid using a uterine manipulator in addition to utilizing the 3^rd^ RA to pull the uterine horn this study adopted. Mabuchi et al. (34) invented a new uterine manipulation device, a U-traction consisting of a 65-mm half-curved cutting needle with a 2.5-mm polyester tape, to ease uterine manipulation of LRH in 8 IB1 cervical cancer patients. Results indicated that no intraoperative complications occurred. Moreover, Xu et al. (35) implemented ameliorative surgical techniques *via* round ligament suspension and vaginal purse-string suture in laparoscopic radical trachelectomy, totaling 12 patients without intraoperative or serious postoperative complications to overcome tumor cell spillages. Recently, Kanao et al. conducted the no-look, no-touch (NLNT) method in LRH (36), which created the vaginal cuff, then manipulated the uterus by using the forceps through the trocar placed in the posterior vaginal fornix and handling the thread around the uterine body, and exposed the paracervical tissue by suspension technique. The surgery and oncological results indicated that the NLNT method made LRH proceed smoothly without worsening the oncological outcomes (37, 38). Also, Meng et al. (17) and Bo et al. (39) performed vaginal ligation under the cervical cancer lesion and removed the vagina below the ligation line without using a uterine manipulator during LRH. The two studies reported the short-term complications of 22 and 8 patients who did not use a uterine manipulator, respectively, indicating no complications such as perioperative vascular injury, pelvic injury, and abdominal organ injury. Moreover, patients in the study by Bo et al. (39) survived without recurrence with a median follow-up of 6 months. The aforementioned two studies by Meng et al. (17) and Bo et al. (39) did not contain a control group. By contrast, we added the control group of 2-RA RRH that used a uterine manipulator conventionally. Meanwhile, we obtained a larger sample size and more comprehensive perioperative indicators.

As we gain more experience, we find 3-RA RRH has some potential merits. One of the advantages is that the 3^rd^ RA can rotate 360 degrees due to its’ human-like wrist structure, which can expose the surgical field of vision while ensuring the lowest injury. Another advantage of the 3-RA RRH is reducing the dependence on surgical assistants, usually residents accepting professional training and were in charge of tissue traction, suction, irrigation, and specimen removal during the operation.

The limitation of this study is its essential retrospective attribute. Firstly, selection bias may be introduced as a retrospective study because the surgical method is not random but evaluated by the surgeon and referred to the patients’ subjective willingness. However, there is no significant statistical difference in important baseline clinicopathological features that may influence the outcomes of the operation. Secondly, 3-year or 5-year survival analysis was delayed because of limited follow-up time; thus, only mortality and recurrence rates were obtained till March 2023. In the future, we will observe the patients’ 3-year or 5-year OS and DFS in the subsequent work to illustrate the further prognosis results. Meanwhile, an increased number of RAs could bring the disadvantage of an increase of more surgical scars and postoperative pain, but the increased pain and disfigurement were not accessed in detail.

We obtained the following conclusion from our study. First, the perioperative efficacy of the 3-RA RRH group was not only non-inferior to or somewhat even superior to that of the 2-RA RRH group, especially when the volume of 3-RA RRH cases exceeded 24. Second, the incidence of postoperative anemia in the 3-RA RRH group is lower than that of the 2-RA RRH group, which may be influenced by less blood loss in the 3-RA RRH group. We assumed that the replacement of a uterine manipulator by the 3^rd^ RA to pull the uterus was the main reason for better surgical outcomes in the 3-RA RRH group.

In conclusion, this study shows that adopting the 3^rd^ RA rather than a uterine manipulator to manipulate the uterus in the 3-RA RRH group is comparable to the 2-RA RRH group, and the short-term perioperative results of 3-RA RRH are slightly better. However, the long-term complications associated with surgery and oncologic outcomes need to be further explored in additional prospective randomized clinical trials.

## Data availability statement

The original contributions presented in the study are included in the article/**Supplementary Material**. Further inquiries can be directed to the corresponding author.

## Ethics statement

The studies involving human participants were reviewed and approved by the Research Ethics Committee of Guangxi Medical University First Affiliated Hospital 2021(KY-E-176). The patients/participants provided their written informed consent to participate in this study.

## Author contributions

XL, HH and YL contributed equally to the research. XL and HH wrote the first draft of the manuscript. SC and JZ performed the data collection. YL, HH, SC and LW analyzed data. HL and BY provided figures. JF, JY, HZ and YL critically revised the paper. JF designed research and provided funding support. All authors contributed to the article and approved the submitted version.
